# A Rare Case of Postoperative Encephalopathy in Twin

**DOI:** 10.7759/cureus.37610

**Published:** 2023-04-15

**Authors:** Chenan A Huang, Caroline Casey, Hussam Ismael

**Affiliations:** 1 Internal Medicine, University of Central Florida College of Medicine, Orlando, USA; 2 General Surgery, Orlando Veterans Affairs Medical Center, Orlando, USA; 3 Surgery, University of Central Florida College of Medicine, Orlando, USA

**Keywords:** anesthesia sensitivity, antihypertensive sensitivity, twin, sepsis associated encephalopathy, hypertensive encephalopathy, dialysis disequilibrium syndrome, uremic encephalopathy, hypoxic/anoxic ischemic encephalopathy

## Abstract

The clinical picture of encephalopathy invites a broad differential with multiple etiologies. It is with judicious history, hospital course, lab testing, and imaging that the ultimate cause is identified. We present a unique case of identical twins who share a similar clinical presentation of postoperative encephalopathy. The striking similarities in both twins suggest a genetic component requiring further research to identify patients who are genetically predisposed.

## Introduction

There are many etiologies of postoperative encephalopathy, including but not limited to hypoxic-ischemic, hypertensive, uremic, and septic encephalopathies. We present a rare case of postoperative encephalopathy in identical twins. The twins had a similar hernia operation, and both developed postoperative encephalopathy with a similar recovery. Their nearly identical clinical decline and recovery support the theory of genetic susceptibility to developing encephalopathy. There is some literature on mechanisms underlying various etiologies of encephalopathy. For example, at the molecular level, genetic factors that alter mitochondrial metabolism can increase blood-brain barrier (BBB) permeability and subsequently increase the susceptibility to septic encephalopathy in murine models [1]. On the clinical scale, variations in patient susceptibility to hypertensive encephalopathy raise the question of genetic components involved in endothelial regulation, cerebral vascular autoregulation, and BBB disturbance [2]. Although mechanisms for encephalopathy have been explored, there is still a lack of research on genetic factors that may predispose patients. In contrast, twin and genetic studies quantifying the role of genetics in metabolic conditions, including diabetes mellitus, obesity, and hypertension, have shed light on those disease processes [3-5]. Similarly, genetic predisposition to neurologic diseases, including Alzheimer’s disease and stroke, has improved understanding of those processes [6-8]. To our knowledge, this is the first report of postoperative encephalopathy in identical twins. We believe that further research is required to determine if there is a genetic susceptibility to developing postoperative encephalopathy. Identifying those who are genetically susceptible can help with prevention or early detection and management of this problem, leading to improved outcomes.

## Case presentation

**Figure 1:**
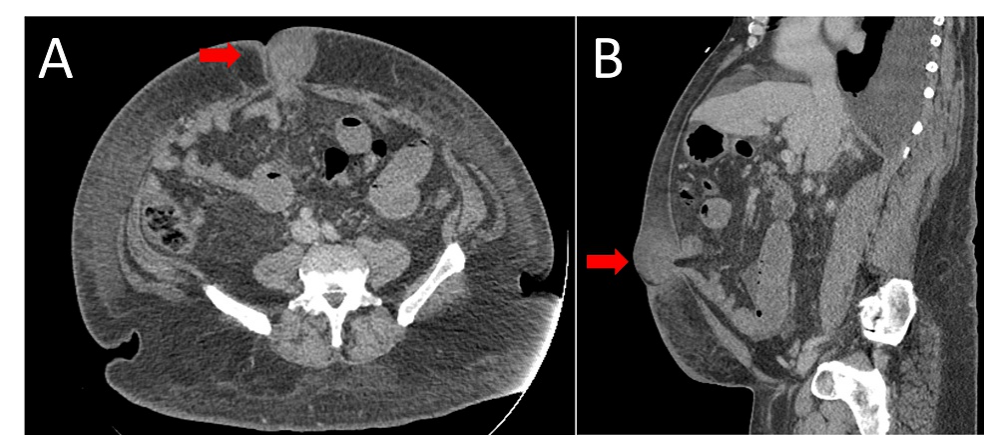
CT abdomen without contrast demonstrating umbilical hernia denoted by red arrows. 1A: Axial view; 1B: Sagittal view.

A 49-year-old morbidly obese male with untreated decompensated congestive heart failure, uncontrolled hypertension, and chronic renal insufficiency stage III presented with symptoms, signs, and radiologic findings consistent with an incarcerated or strangulated periumbilical hernia containing small bowel. On presentation, the patient’s vitals were significant for hypertension at 168/77. The patient underwent urgent exploratory laparotomy and was found to have a segment of ischemic small bowel within the hernia (Figure 1). A small bowel resection with primary anastomosis was performed, and the hernia defect was repaired. The patient was transferred to the ICU, where he was extubated with no focal neurological or motor deficits. On postoperative day 1 (POD#1), the patient had a Glasgow Coma Scale (GCS) of 15 and was interactive with the medical staff. His blood pressure was 165-190/65-70 on hydralazine. He developed acute on chronic renal insufficiency with a decrease in urine output.

**Figure 2:**
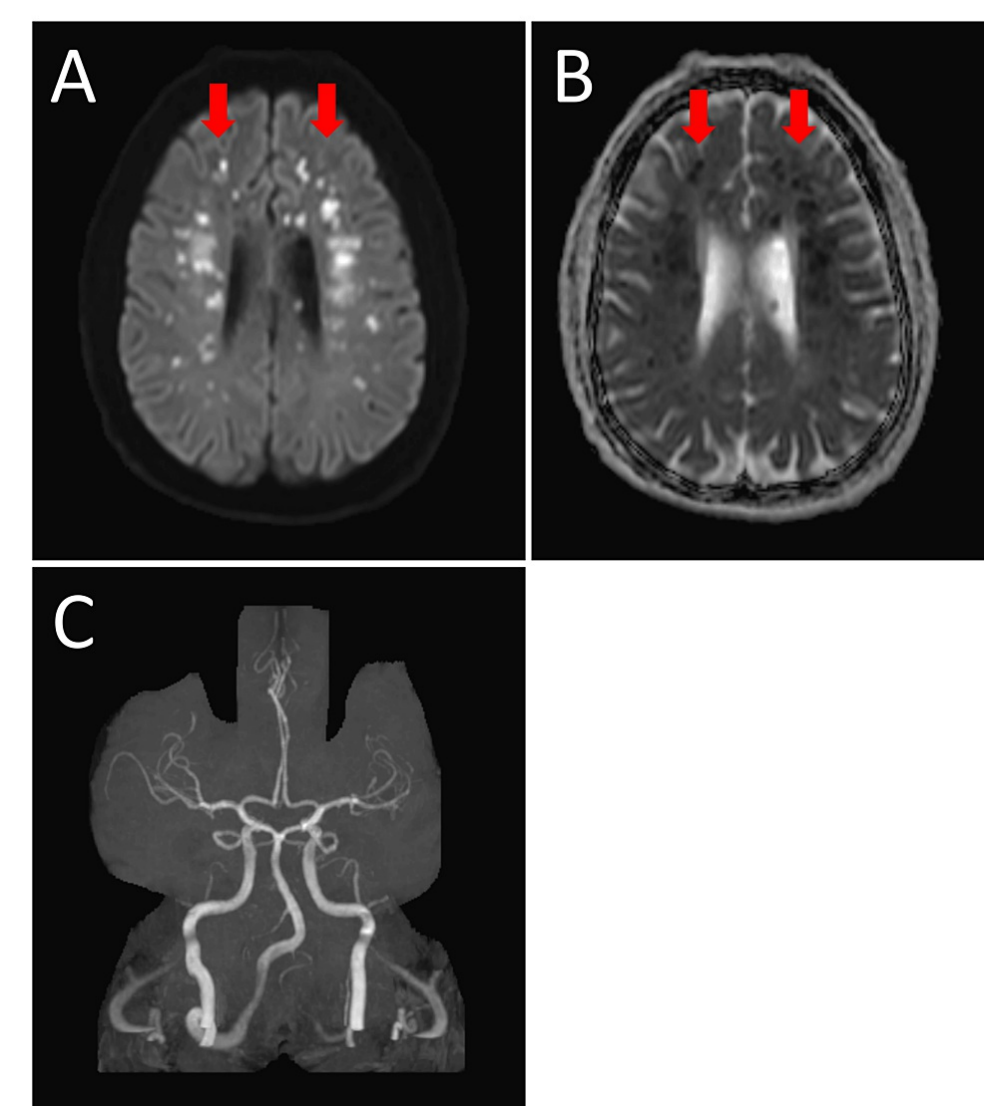
MRI head without contrast demonstrating multiple foci of infarction indicated by red arrows and MRA of the circle of Willis showing no evidence of large vessel occlusion. 2A: Axial view of diffusion-weighted imaging (DWI) MRI; 2B: Axial view of T2-weighted MRI; 2C: 3-D MRA. MRA: Magnetic resonance angiography.

On POD#2, the patient experienced abrupt mental decline, with a GCS of 9. CT of the patient’s head revealed no acute intracranial hemorrhage, mass effect, edema, midline shift, or extra fluid collections. However, some chronic processes were noted, including low-density changes in the periventricular white matter compatible with mild small vessel disease, vascular calcifications, and chronic ischemic changes. Normal lab values excluded electrolyte abnormalities, hypoxia, hypercapnia, and hypoglycemia as etiologies for the patient’s altered mental status. Labs were only remarkable for BUN 34, creatinine 2.7, albumin 2.6, and total bilirubin 2.33. The patient was reintubated on POD#11 as he could not maintain his airway. His GCS was 3 off sedation. He continued requiring respiratory support; tracheostomy and gastrostomy tubes were eventually placed. His renal function continued to decline, leading to fluid overload and uremia, with a BUN reaching 121, requiring hemodialysis. The patient’s mental status varied between GCS 3 and 6 for the next 45 days of his ICU stay. Initial MRI without contrast (Figures 2A-2B) of his head revealed multiple, non-specific foci of acute to subacute infarcts of the bilateral cerebellum and cerebral hemispheres suspicious for an embolic event. However, lower extremity ultrasound (US), carotid US, and magnetic resonance angiography (MRA) (Figure 2C) were negative for stenosis or large vessel occlusion. Thus he was managed conservatively with antihypertensives and hemodialysis. As embolic sources of the MRI findings were excluded, hypoxic-ischemic encephalopathy became our leading diagnosis. His GCS eventually improved to 8-10, at which point he was transferred to a long-term care facility on ventilator support and received tube feeds via his gastrostomy tube. Eventually, our patient regained consciousness. After several months of rehab and living in a long-term care facility, he regained some neurologic and motor functions. He is currently able to sit with assistance and follow basic commands.

Collateral obtained from the parents of this patient revealed striking similarities to his identical twin brother, who also suffered a decline in mental status following an incarcerated/strangulated umbilical hernia repair. The twin brother presented when he was 47 with a history of uncontrolled hypertension and an incarcerated hernia requiring bowel resection. However, he fell into a coma immediately postoperatively, without a lucid period. The twin required an approximately 45-day ICU stay with a similar course. He required intubation, tracheostomy, gastrostomy and eventually developed renal failure requiring hemodialysis. After regaining consciousness, he was transferred to inpatient care for another 45 days. After three months postoperatively, he was discharged to a long-term care facility where he currently lives. He regained some function and is able to talk and walk with assistance.

## Discussion

Some etiologies of mental status decline that fit with the patient’s history, treatment course, and clinical presentation include hypoxic-ischemic encephalopathy (HIE), hypertensive encephalopathy (HE), uremic encephalopathy (UE), dialysis disequilibrium syndrome (DDS), and sepsis-associated encephalopathy (SAE). Although some characteristics of each etiology were seen in this patient, the equivocal initial MRI resulted in difficulty in choosing a management course. Additionally, the labs did not point to a specific etiology of encephalopathy. The purpose of this review is to provide an initial differential in a patient with an encephalopathy of unclear etiology and provide pearls and pitfalls of diagnostic testing and management. In addition to working through clinical decision-making, the presentation of identical twins meeting strikingly similar post-surgical outcomes raises the question of a genetic component at play. Twin and genetic studies have quantified the genetic component of metabolic conditions, including diabetes mellitus, obesity, and hypertension [3-5]. These factors, however, do not fully account for the postoperative decline and recovery of the twins in this case. Genetic predilection in neurobiology has been explored for Alzheimer’s disease and stroke, for example, but no studies have been conducted on genetic factors in acquired encephalopathies [6-8]. Mechanistically, the role of BBB permeability, neurovascular autoregulation, and mitochondrial metabolism in developing encephalopathy has been suggested. However, there is a paucity of research expanding on the underlying genetic component involved in those mechanisms [1,2,9,10]. To our knowledge, this is the first report of postoperative encephalopathy in identical twins.

Hypoxic-ischemic encephalopathy

HIE is typically seen in newborns with birth complications leading to global cerebral hypoxia and ischemia. In adults, HIE can result from cardiac arrest, airway obstruction, decreased inspired oxygen content, hanging, drowning, carbon monoxide poisoning, or cyanide poisoning [11]. Although HIE is classically thought of as affecting watershed regions, those are not the only areas impacted. The cortex (frontal, parietal, and occipital), as well as deep brain structures (thalamus and basal ganglia), can also be affected [11]. Additionally, injury to the cerebral cortex, cerebellum, neostriatum, hippocampus, and brain stem were correlated with unfavorable outcomes [11,12]. In addition to imaging studies, somatosensory evoked potentials (SSEP) can be used as a prognostic tool. SSEPs are measured via brain activity recorded from the somatosensory cortex after median-ulnar nerve stimulation [13]. Some drawbacks of SSEP recording include a 1-2% false-positive rate and finding an appropriately skilled operator who can identify artifacts, including electrical interference, muscle activity, sedatives, and temperature [13]. Targeted temperature management (TTM) is the process of externally controlling a patient’s temperature for therapeutic purposes. TTM at 32ºC-36ºC is currently recommended by the 2015 American Heart Association Guidelines Update for cardiopulmonary resuscitation and emergency cardiovascular care for patients who are comatose with a return of spontaneous circulation after cardiac arrest [13]. The temperature has a critical role in the neurological recovery after HIE. The wide range of therapeutic temperatures helps adjust for patient condition and morbidity, including bleeding risk and pre-TTM body temperature [13]. Hyperthermia in comatose patients is associated with worsened outcomes, so leaving TTM devices in place to prevent hyperthermia after therapeutic hypothermia is appropriate [13]. Seizures, non-convulsive status epilepticus, and other epileptiform activity may be seen in up to 12%-22% of HIE patients [13]. As prolonged epileptiform discharges are associated with brain damage in other situations, antiepileptic administration is imperative in the immediate comatose period. However, electroencephalogram (EEG) monitoring is still required, as prophylactic thiopental or phenobarbital do not provide any outcome benefits [13]. For this patient, anesthesia for emergent exploratory laparoscopy complicated blood pressure management, resulting in brief systolic pressures in the 120-130 mmHg range. This brief period of relative hypotension complicated the clinical picture. Postoperatively, blood pressures were stabilized to 165-190/65-70 mmHg with a slow reduction to the normotensive range for days. The culprit of our patient’s HIE likely lay in aggressive blood pressure control in the preoperative and intraoperative periods. Due to the patient’s history of uncontrolled hypertension, titrating his blood pressure proved difficult, especially compounded by the emergent nature of his presentation. Clinically, the patient’s lucid interval and initial MRI findings made diagnosis challenging. In the absence of a clear etiology but with high clinical suspicion, continuous EEG monitoring with SSEP and TTM could have improved this patient’s outcome. 

Hypertensive encephalopathy

HE is defined as acute neurological dysfunction associated with acute and severe hypertension. Although no cutoff pressures have been identified, blood pressures are typically over 220/120 mmHg [14,15]. Severe increases in blood pressure can be categorized as accelerated or malignant hypertension. Accelerated hypertension is identified by retinal changes, including exudates, hemorrhages, arteriolar narrowing, and spasms but without papilledema [14]. Malignant hypertension is considered a more severe form, distinguished by papilledema [14]. Clinical manifestations of accelerated or malignant hypertension include visual disturbances, headache, altered mental status, seizure, and, if untreated, coma and death [1,14,15]. Renal damage may result in azotemia, proteinuria, and anemia [14]. Worsening clinical symptoms may signal progression to HE, which can be rapid, especially when complicated by renal insufficiency [14]. History and physical exams are typically enough to diagnose and treat HE, but it is important to note that the absence of papilledema does not rule out HE [14,15]. Guidelines on blood pressure management are lacking, but the consensus of HE management suggests an immediate reduction of blood pressure to no more than 20-25% of a patient’s typical blood pressure and a progressive reduction of blood pressure to normotensive values over 24 hours [14,15]. No single antihypertensive has been proven to be superior and typical agents include IV labetalol, nicardipine, and nitroprusside infusions [15]. On presentation, this patient’s blood pressure was in the 200/100 mmHg range, raising suspicion for HE. Blood pressure control was prioritized, but his typical blood pressure and the duration of marked hypertension were both unknown. In addition to markedly elevated blood pressure, his pre-existing renal insufficiency increased his risk for HE. Although HE was suspected, the course of his mental status change does not reflect the chronicity of his blood pressure, and MRI findings were not consistent with HE.

Uremic encephalopathy

UE develops as a result of acute renal dysfunction leading to the accumulation of uremic toxins altering the balance of excitatory and inhibitory neurotransmitters [16]. Clinically, UE presents with mental status changes, including mild confusional states, emotional changes, cognitive/memory deficits, confusion, delirium, psychosis, seizures, and coma. UE is often associated with movement disorders, such as fine action tremors, asterixis, and hyperreflexia [16]. Treatment aims to reduce uremic toxins through renal replacement therapy with dialysis and/or renal transplant. Typically, neurological symptoms of UE resolve within days to weeks after initiating dialysis or within days post renal transplant, but mild symptoms may linger. Complications of dialysis include subdural hematoma, intracranial hemorrhage, and Wernicke’s encephalopathy [16]. Subdural hematoma and intracranial hemorrhage are presumably secondary to comorbid hypertension with associated vascular changes in the brain exacerbated by anticoagulation during dialysis [16]. Wernicke’s encephalopathy can occur in any malnourished patient, and patients with chronic kidney disease or end-stage renal disease commonly have suboptimal nutritional status. Causes include increased catabolism, gut microbe dysregulation, and hormonal derangements [17]. The full triad of neurological symptoms, ataxia, and ocular abnormalities are rarely all present in Wernicke’s encephalopathy, so treatment is typically initiated with high clinical suspicion. This patient’s BUN was moderately elevated early during his stay but increased rapidly due to acute on chronic renal failure. His relative hypotension during surgery may have incited the rise in BUN. However, the rise of BUN to 121 mg/dL was well after his decline in mental status. The chronicity of symptoms does not coincide with the rise in BUN, making UE an unlikely cause. Additionally, dialysis effectively lowered his BUN but did not improve his mental status. The resolution of this patient’s azotemia did not result in clinical improvement, which may indicate the development of DDS, which will be discussed later. Although it is unlikely uremia caused this patient’s initial decline, DDS may have contributed to his prolonged recovery.

Dialysis disequilibrium syndrome

DDS is a cause of altered mental status secondary to rapid changes in metabolites after dialysis. Rapid decreases in peripheral BUN leading to an osmotic gradient across neurons and increased neuronal permeability to water and urea result in large intracellular water shifts and subsequent cerebral edema [9]. The clinical presentation of DDS can include acute onset of headache, nausea, vomiting, disorientation, a confusional state, seizures, and coma [9]. DDS is more common in children, in patients with a history of head injury, subdural hematoma, stroke, and malignant hypertension, and in patients with conditions predisposing to cerebral edema [9]. DDS can be prevented by decreasing hemodialysis length to 2-3 hours, dialyzing daily, and generally reducing hemodialysis efficacy during the first sessions [9]. Other strategies to minimize DDS include increasing dialysate sodium (2 mEq/L of sodium is equivalent to approximately 11 mg/dL of BUN) and administration of other osmotically active substances (glucose or mannitol). These strategies minimize the urea and overall osmotic gradient to limit cerebral edema [9]. DDS may have contributed to the prolonged coma of this patient, but the diagnosis was confounded by the patient’s comatose state prior to renal failure and subsequent dialysis. The patient’s BUN was rapidly lowered from 121 mg/dL within the first dialysis session, but as the patient’s GCS was 3 at the time, no diagnosis could be made.

Sepsis-associated encephalopathy

SAE causes neurologic dysfunction ranging from delirium to coma and is secondary to severe systemic infection without CNS involvement. Many neurologic symptoms are multifactorial in nature and can include structural lesions (cerebral ischemia, hemorrhages, and micro-abscesses), altered cerebral microcirculation, increased BBB permeability, and increased inflammatory mediators [2,10]. SAE is found in over 50% of patients with sepsis and carries about a 70% mortality rate from multiorgan failure in patients with severe SAE [10]. Patients with SAE have severe infections with features of severe sepsis or system inflammatory response syndrome (SIRS) [10]. SIRS is defined by body temperature above 38°C or below 36°C, hyperventilation (respiratory rate above 20 breaths per minute or arterial carbon dioxide below 32 mmHg), and WBC count above 12,000 or below 4,000 cells/μL [18]. Staphylococcus aureus, Enterococcus faecium, Acinetobacter spp., Pseudomonas aeruginosa, and Stenotrophomonas maltophilia are the most commonly identified species in biliary or intestinal infections, which have a greater risk of SAE [10]. Early detection of sepsis and treatment with broad-spectrum antibiotics is key to preventing SAE, with delirium often being the first sign of infection [10]. In addition to antibiotics, EEG monitoring should be employed to detect non-convulsive seizures and inform antiepileptic administration. In contrast to other seizure etiologies, for the treatment of seizures as a result of SAE, lorazepam is inferior. In a placebo-controlled trial comparing dexmedetomidine and lorazepam in patients with sepsis, patients treated with dexmedetomidine had more encephalopathy-free days, shorter time on the ventilator, and lower mortality than those treated with lorazepam [10]. Although this patient presented with bowel strangulation and tissue necrosis, he did not develop signs of local or systemic infection. Only preoperative antibiotics were administered, and the patient did not require additional antibiotics.

## Conclusions

In evaluating and managing a complex patient who develops postoperative encephalopathy, it is essential to rule out a broad differential systematically. We present a unique case of identical twins with strikingly similar presentations and postoperative outcomes, suggesting a genetic role. Both patients developed postoperative encephalopathy and had a prolonged and nearly identical recovery. Further research is needed to identify patients genetically predisposed to postoperative encephalopathy.
